# Exosomal LGALS9 in the cerebrospinal fluid of glioblastoma patients suppressed dendritic cell antigen presentation and cytotoxic T-cell immunity

**DOI:** 10.1038/s41419-020-03042-3

**Published:** 2020-10-22

**Authors:** Ming Wang, Yang Cai, Yong Peng, Bo Xu, Wentao Hui, Yugang Jiang

**Affiliations:** 1 Department of Neurosurgery, The Second Xiangya Hospital of Central South University, 410011 Changsha, Hunan China; 2 Department of Neurosurgery, The First Affiliated Hospital of Xi’an Jiaotong University, 710000 Xi’an, Shanxi China; 3 Department of Biochemistry and Molecular Biology, Nanjing Normal University, 210000 Nanjing, Jiangsu China

**Keywords:** Tumour immunology, Prognostic markers

## Abstract

Glioblastoma multiforme (GBM) is highly invasive, with a high recurrence rate and limited treatment options, and is the deadliest glioma. Exosomes (Exos) have attracted much attention in the diagnosis and treatment of GBM and are expected to address the severe limitations of biopsy conditions. Exos in the cerebrospinal fluid (CSF) have great potential in GBM dynamic monitoring and intervention strategies. Here, we evaluated the difference in the proteome information of Exos from the CSF (CSF-Exos) between GBM patients and low-grade glioma patients, and the correlations between GBM-CSF-Exos and immunosuppressive properties. Our results indicates that GBM-CSF-Exos contained a unique protein, LGALS9 ligand, which bound to the TIM3 receptor of dendritic cells (DCs) in the CSF to inhibit antigen recognition, processing and presentation by DCs, leading to failure of the cytotoxic T-cell-mediated antitumor immune response. Blocking the secretion of exosomal LGALS9 from GBM tumors could cause mice to exhibit sustained DC tumor antigen-presenting activity and long-lasting antitumor immunity. We concluded that GBM cell-derived exosomal LGALS9 acts as a major regulator of tumor progression by inhibiting DC antigen presentation and cytotoxic T-cell activation in the CSF and that loss of this inhibitory effect can lead to durable systemic antitumor immunity.

## Background

Extracellular vesicles (EVs) are lipid membrane-bound nanoparticles with a diameter of 40 to 1000 nm, and as EVs are carriers for intercellular communication, almost all cells release EVs^1,2^. EVs play important roles in the development and maintenance of cancer and can be used as potential targets for diagnosis and intervention^3^. EVs have high heterogeneity, which is related to the diverse cell populations in the microenvironment. They carry specific markers, proteins, nucleic acids or metabolites involved in cross-cell regulation^3^. EV particle size also seems to determine function. Among EVs, exosomes (Exos) with a particle size of approximately 100 nm have attracted much attention, especially in a variety of malignant tumors^3^. Some studies have shown that cancer cells can assemble special proteins in Exos so that the expression pattern of the cancer cells is no different from that of normal cells, which often causes researchers to overlook the lethal targets recognition of some nondifferentially expressed^4^.

Gliomas are inherent brain tumors. According to the 2016 central nervous system tumor classification revised by the World Health Organization (WHO), adult gliomas are classified into grades II to IV^5^. WHO grade II (GII) tumors predominantly have an oligodendroglial morphology with a good prognosis and are often accompanied by IDH mutation and 1p/19q codeletion. WHO grade III (GIII) tumors usually have an astrocytoma morphology, and patients have a moderate survival rate. WHO grade IV tumors (glioblastoma multiforme, GBM) often have a poor prognosis with gain of chromosome 7 and loss of chromosome 10. There is no cure for GBM, the median survival time is 14 months, and the 5-year survival rate is <3%^6,7^. GBM tumors express immunosuppressive molecules, such as PD-L1, IDO, or IL-10, and exhibit reduced expression of HLA-A molecules to limit the self-presentation of tumor antigens and ultimately escape immune surveillance^8^. In addition, the GBM immune environment lacks effective T-cell infiltration^9^ and exhibits excessive accumulation of M2-like myeloid cells and regulatory T cells^10^, which together lead to strong immunosuppression in GBM; on the other hand, this microenvironment allows GBM patients to benefit from immunotherapeutic strategies. Dendritic cells (DCs) can traffic to the deep-cervical lymph nodes of the brain and present antigens to promote a cytotoxic T-cell response, although this process might be abolished in the context of systemic immunosuppression^8^.

The location of GBM is usually sensitive and difficult to access, which makes the biopsy approach used to detect disease progression meaningless. Cerebrospinal fluid (CSF) is a colorless, transparent liquid that exists in the ventricles and subarachnoid space. It plays a role as a lymphatic fluid in other parts of the body by removing metabolites and inflammatory exudates. As the CSF directly surrounds the brain, it can indirectly reflect the immune environment of GBM. In addition, compared with fluid collected by surgical suction, which causes destruction of tumor tissue and the microenvironment, CSF is a good material for noninvasively studying the GBM microenvironment.

In this study, we collected CSF from GII–III and GBM patients, detected the differences in the immune cell composition and Exo proteomics, and used cell and animal models to screen potential molecules suitable for the diagnosis and treatment of GBM.

## Materials and methods

### CSF specimens and cohorts

The content of this study involving human experiments was performed in accordance with the guidelines of the “Helsinki Declaration” and “European Declaration of Human Rights”. The CSF of patients was prospectively collected within 24 months (beginning in October 2016). Informed consent was obtained from the control group subjects (who were excluded if they had tumors or central nervous system disease), GII–III patients and GBM patients. The experimental protocol was carried out with the approval of the Ethics Review Committee of First Affiliated Hospital of Xi’an Jiaotong University School of Medicine. Primary WHO grade IV GBM (*n* = 7) and GII–III (astrocytoma (*n* = 2), and oligodendroglioma (*n* = 5)) cases were confirmed histopathologically. All GBM tumors were IDH wild-type, and all grade II–III lesions carried IDH1 mutations. Supplementary Table S2 provides detailed information for all subjects. For the collection of CSF samples, a 22-G×3 1/2-inch noninvasive Sprotte spinal needle (Pajunk, Germany) was used to collect 2 mL of CSF into a sterile polyacrylamine centrifuge tube and centrifuged at 2000 × *g* for 15 min to separate the cells from the supernatant.

### Commercial cell lines

The mouse glioma cell line GL261 (KCB 200770YJ), the human malignant brain astroglioma U87MG (KCB2011101YJ) and U118 MG(KCB201302YJ) were purchased from the Kunming Cell Bank of the Chinese Academy of Sciences. Primary human astrocytes (HA) was purchased from the Sciencell Research (SanDiego, CA, USA). GL261 and U118 MG cells were cultured in Dulbecco’s modified Eagle’s medium (DMEM; Gibco, Grand Island, NY, USA) containing 10% fetal calf serum (FCS; Gibco) and 1% penicillin–streptomycin (Life Technologies, Gaithersburg, MD) at 37 °C and 5% CO_2_. U87 MG cells were cultured in Minimum Essential Medium (MEM) (Gibco) containing 1% non-essential amino acids (NEAA) (Gibco), 10% fetal bovine serum (Gibco) and 1% penicillin–streptomycin (Life Technologies) at 37 °C and 5% CO_2_.

### Production of human DCs and T cells from PBMCs

Relatively homogeneous functionally mature DC populations can be generated from CD14 + blood monocytes by incubation with appropriate cytokines^11^. Whole-blood samples were obtained from blood center of ChangSha (HuNan, China). Briefly, blood samples were placed in vacutainer tubes (Becton Dickinson, UK) containing EDTA, and peripheral blood mononuclear cells (PBMCs) were isolated using Histopaque-1077 (Sigma, Dorset, UK). PBMC were frozen in a mixture containing 90% autologous plasma and 10% DMSO and stored in a liquid nitrogen refrigerator. PBMC suspension cells are used to induce T-cell differentiation, and adherent cells are used to induce DCs differentiation. CD14 + monocytes were isolated from the PBMCs adherent cells using a MACS system (Miltenyi Biotech, Bergisch Gladbach, Germany) according to the manufacturer’s protocol. In total, 5 × 10^6^ CD14 + monocytes per well were seeded in 12-well plates (Corning Inc., Costar, NY, USA) containing 0.3 g/L l-glutamine (Sigma), 5% fetal bovine serum (Gibco), and 1% penicillin–streptomycin (Life Technologies) in RPMI 1640 medium (referred to as complete medium, 5% CM). After a 2-h incubation at 37 °C, the cells were washed gently with 5% CM to remove nonadherent cells. PBMCs were cultured with cytokines to induce differentiation into DCs^12^; specifically, 800 U/mL GM-CSF (R&D Systems, Abingdon, UK) and 500 U/mL IL-4 (R&D Systems) in 5% CM were used. The PBMCs were resuspended at a density of 1 × 10^6^ cells/mL in 5% CM and seeded in tissue culture flasks. Fresh 5% CM containing GM-CSF and IL-4 was added to the culture on day 3. On day 5, 5% CM containing 100 U/mL TNF-α (R & D Systems), GM-CSF and IL-4 was added. On day 8, the cells were resuspended by vigorous pipetting to disrupt cell aggregates and washed to remove the semiadherent cells from the culture wells. For T cells, after thawing PBMC, they were treated with DNase I (Sigma) at 200 U/mL at 37 °C for 20 min, and then cultured in a humid incubator at 37 °C and 5% CO_2_ for 1 hour. In all, 20 ng/mL TGF-β, IL-10, and IL-4 (both Sigma), 25 ng/mL MCSF (Gemini Biosciences) were used to induce nonadherent PBMC differentiation.

### Antibodies, flow cytometry, and western blot analysis

For the determination of myeloid and lymphoid cells percentage, 2 × 10^5^ cells centrifuged from human CSF, mice CSF or CM were resuspended in 0.5% bovine serum albumin (BSA) in phosphate-buffered saline (PBS), blocked with anti-human CD16/32 FCS for 1 h. myeloid cells were detected with fluorescently labeled antibodies against CD45, CD11B, CD11C,LY6G, LY6C, CD11B and HLA-DR (MHC II) (eBioscience, SanDiego, CA, USA) and lymphoid cells were detected using fluorescently labeled antibodies specific for CD45, CD11B, CD11C, CD4, and CD8 (eBioscience). For detection of functional or intracellular proteins by flow cytometry, 0.5 × 10^5^ DCs or T Cells were treated and permeabilized with an intracellular immobilization buffer (Thermo Fisher) and then blocked with 0.5% BSA in PBS for 2 h. The cells were incubated overnight at 4 °C with antibodies against HLA-A, CD40, CD86, TAP1, KI67, INF-γ Gazm B, and LGALS9 (ABclonal Technology, Wuhan, CHN) and then incubated with a corresponding fluorescently labeled secondary antibody for 2 h at room temperature in the dark; a negative isotype control was also utilized. All flow cytometry analyses were performed on a BD FACSCanto II flow cytometer (BD Biosciences), and the results were analyzed using FlowJo software (TreeStar Inc.). To detect protein expression by western blot^13^, cells were collected and used to generate a cell lysate (Beyotime), 20 μL of which was heated at 100 °C for 15 min and then loaded onto an sodium dodecyl sulfate polyacrylamide gel electrophoresis gel. Electrophoresis was carried out at 90 V (30 min) and 120 V (80 min). The proteins were transferred to a nitrocellulose membrane (Beyotime) at 0.38 A (100 min) using a wet transfer method, and the nitrocellulose membrane was blocked with 5% skim milk (90 min), washed three times with PBS (3 min each) and incubated with a diluted primary antibody overnight at 4 °C. After washing with TBST three times (3 min each), the membrane was incubated with a corresponding HRP-labeled secondary antibody for 1 h at room temperature, developed using a DAB horseradish peroxidase chromogenic kit (Beyotime) and visualized using a chemiluminescence imaging system (Bio-Rad).

### Separation and characterization of EVs and liquid chromatography–mass spectrometry analysis of protein cargo

EVs were separated from the collected CSF or cell culture medium by centrifuging at 300 × *g* for 10 min and 2000 × *g* for 15 min, and then the supernatant was passed through a series of injection filters with a gradually decreasing pore-size (Sigma). The pore sizes were 1, 0.4, and 0.1 μm. Nanosight (Malvern Panalytical, Malvern, UK) was used for EV counting and particle size detection, video recordings were analyzed using NTA 3.1 software, and each sample was imaged three times. EVs were imaged using TEM, and separated EVs were suspended in 30 μL of PBS at 4 °C. The eluted EVs were adsorbed onto a Formvar carbon-coated electron microscope grid and imaged at 80 kV under a Tecnai G 2 Spirit BioTWIN microscope. Liquid chromatography coupled with tandem mass spectrometry (LC-MS) was used to detect EV cargo. EVs were dissolved in tetraethylammonium bicarbonate containing 0.2% proteolytic denaturant Rapigest (Waters Corporation, MA, USA) at 50 mM. After incubating at 95 °C for 5 min, the EVs were placed on ice, sonicated for 30 s to release EV proteins, incubated at 60 °C in 12 mM tris (2-carboxyethyl) phosphine for 60 min, and then incubated at room temperature in 50 mM iodoacetamide for 60 min in the dark to reduce and alkylate the proteins. The samples were incubated overnight with 4% trypsin at 37 °C to obtain cleaved peptides. The peptides were acidified with 50% TFA, and the precipitate was removed by centrifugation at 13,000 × *g* for 15 min at 4 °C. The peptides were eluted using 70% ACN/0.1% TFA on a 1-cc HLB solid-phase extraction column (Waters Oasis, Massachusetts, USA), followed by centrifugal vacuum drying and protein quantification. The peptide powder was dissolved in 3% ACN/0.1% TFA, and the peptides were analyzed using a Q-Exactive hybrid quadrupole-orbitrap mass spectrometer (Thermo Scientific). The mobile-phase buffer consisted of A: 0.1% TFA and B: 80% ACN and 0.1% TFA. The peptides were eluted using a linear gradient of 5% B to 42% B at a constant flow rate of 250 nL/min within 120 min. The mass spectrometer scanned with 350–1550 m/z, and sample injection was repeated three times. Mass spectrometry results using Mascot (Matrix Science, London, UK; version 2.4.0) referred to the SwissProt database (SwissProt_2018_05). Multiple comparisons were performed using the normalized spectral abundance factor, Student’s *t*-test and the Benjamini–Hochberg correction to determine the abundances of differential proteins. GO analysis was used to annotate the proteins in EVs by determining the enriched biological processes (BP), molecular functions (MF) and cellular components (CC) (DAVID, v7.0)^14,15^.

### CRISPR/Cas9 knockout cell line

This protocol was performed according to frequently used procedures^16^. Target gene sgRNA oligonucleotides (GenScript, Nanjing, China) were cloned into the pLVX-IRES-mCherry vector (Clontech, CA, USA), and two different sgRNA guides were designed for each gene. Lipofectamine 3000 (Thermo Fisher) was used to transfect 1 µg of each plasmid into U87 MG cells, GL-261 cells or DCs. After 48 h, the transfection efficiencies of LGALS9^−/−^, Rab27a^−/−^, and nSMase2^−/−^ U87 MG cells and TIM3^−/−^ DCs were detected by measuring mCherry + fluorescence intensity, and the protein levels in the cells were detected by Western blotting or flow cytometry to determine the knockout efficiency. The sgRNA sequences are shown in Supplementary Table S3.

### Polysome fractionation and RNA isolation

The polysome profiling experiment^17^ was used to detect the efficiency of translation of an mRNA into a protein. In short, HA, U78 MG and U118 MG cells were pretreated with 100 μg/mL cycloheximide (Sigma) for 10 min. Lysis buffer (10 mM Tris-HCl pH 8, 140 mM NaCl, 1.5 mM MgCl2, 0.25% NP-40, 0.1% Triton X-100, 50 mM DTT, 150 μg/mL cyclohexanamide, and 640 U/mL RNasin) was used on ice. The cells were lysed for 30 min, and the supernatant was obtained by centrifugation at 10,000 × *g*. The supernatant was transferred to a 10%–50% sucrose-padded gradient centrifuge tube, and after centrifugation at 35,000 × *g* for 3 h, qPCR was used to detect the mRNA level of LGALS9 in each sucrose layer.

### PCR detection of LGALS9 expression

Total RNA was extracted from HA, U87 MG and U118 MG cells using QIAZOL reagent (Tiangen, Beijing, CHN). In all, 5× FastKing-RT SuperMix (Tiangen) was used for reverse transcription to obtain cDNA, SYBR green (Thermo Fisher) was used on a QuantStudio 3 real-time quantitative PCR system (Thermo Fisher) to quantify LGALS9 mRNA expression, and GAPDH was used as an internal reference. The primers were as follows: LGALS9 forward primer: 5ʹ-GTCTCCAGGACGGACTTCAG-3ʹ, LGALS9 reverse primer: 5ʹ-CGTACCCTCCATCTGGTTCC-3ʹ; and GAPDH forward primer: 5ʹ-GGGTGATGCAGGTGCTACTT-3ʹ, GAPDH reverse primer: 5ʹ-GGCAGGTTTCTCAAGACGGA-3ʹ.

### Obtaining CSF from tumor-bearing mice

C57BL/6J mice were purchased from Charles River (Beijing, China). Immunodeficient NOD-SCID mice were obtained from Modelorg (Shanghai, China). Mice were housed in specific pathogen-free conditions, and 8- to 10-week-old mice were used for tumor experiments. Animal experiments were conducted with the approval of the Animal Care and Use Committee of Nanjing Normal University. Mice that died of nontumor-related causes (for example, fights or infections) were removed, and when the tumor grew to ≥1000 mm^3^ in diameter, the mice were sacrificed. U87 MG WT, U87 MG LGALS9^−/−^, U87 MG Rab27a^−/−^ or U87 MG nSMase2^−/−^ cells (1 × 10^6^) were implanted subcutaneously in mice. On day 14, the mice were euthanized, and the spleen and CSF were collected. Tumor volume was measured every two days and calculated according to the formula: length × width × height × 0.5. For mouse CSF collection, mice were anesthetized with ether anesthesia, and an incision was made from the head to the occipital tuberosity at the midline (4 mm) and then to the shoulder (1 mm), followed by blunt separation. The occipital bone was cut to the atlas muscle with iris scissors to expose the white dura mater, a needle was used to puncture 2 mm between the occipital bone and the atlas, and 2.5 µL of CSF was collected with a micropipette. The LGALS9 expression and immune cell composition of the CSF were detected.

### ELISA and a CCK-8 test

According to the manufacturer’s instructions for the appropriate enzyme-linked immunosorbent assay (ELISA) kit (R & D Systems, Minnesota, USA), the release of IFN-γ and GzmB by T cells was detected. According to the manufacturer’s instructions for cell counting kit-8 (CCK-8; Dojindo, Kyushu, JPN), the cell proliferation of 87 MG cells was tested.

### Statistical analysis

Data were analyzed using one-way analysis of variance or a paired Student’s *t*-test. Statistical analyses and graphical representation of the data were performed using GraphPad Prism 6.0 (GraphPad Inc.) and SPSS version 19.0 (IBM Corp.). *p* < 0.05 was considered to indicate a statistically significant difference.

## Results

### The microenvironment of GBM-CSF is immunosuppressive

**Fig. 1:**
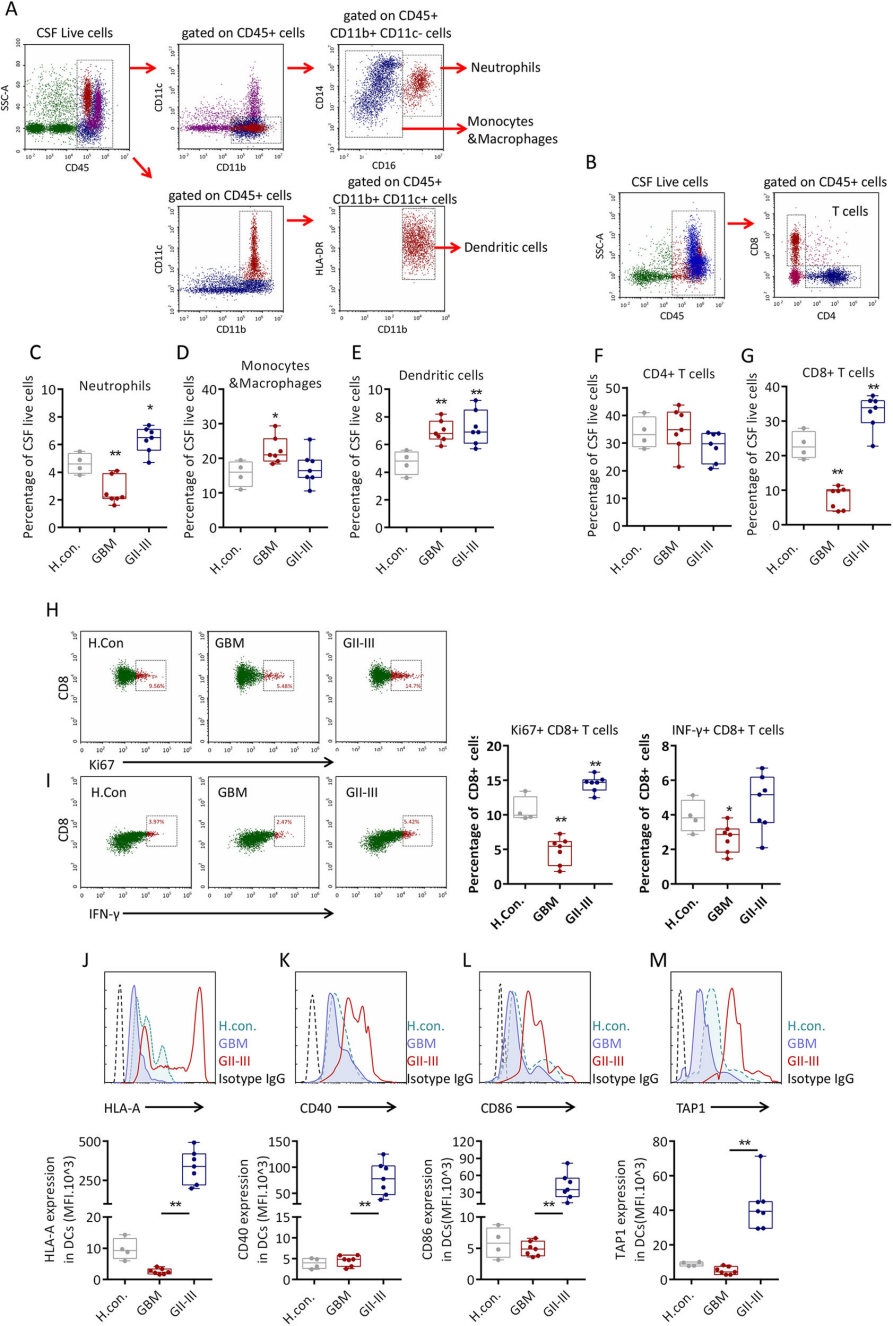
Immunophenotypes of myeloid cells and lymphocytes in the CSF. **a** Representative patterns of human cerebrospinal fluid (CSF) neutrophils, monocytes/macrophages and dendritic cells labeled with different fluorophore-tagged anti-human CD45, CD11B, CD11C, CD14, CD16, and HLA-DR primary antibodies determined by flow cytometry. **b** Representative patterns of human CSF CD4 + T and CD8 + T cells labeled with different fluorophore-tagged anti-human CD45, CD4, and CD8 primary antibodies determined by flow cytometry. **c**–**e** Flow cytometric quantification of the percentages of neutrophils, monocytes/macrophages and dendritic cells in healthy control, GII–GIII patient and GBM patient CSF. **f**, **g** Quantification of the percentages of CD4 + T and CD8 + T cells. **h**, **i** Quantification of the percentages of Ki67 + CD8 + T cells and INF-γ + CD8 + T cells. **j**–**m** Quantification of the mean fluorescence intensities (MFIs) of HLA-A, CD40, CD86, and TAP1 expression in CSF dendritic cells. **h** con (*n* = 4), GII–GIII (*n* = 7), GBM (*n* = 7). Data are shown as the mean ± SD; *p < 0.05 and **p < 0.01 by a *t*-test.

In normal CSF, blood-derived cells are mainly composed of nearly 80% lymphocytes and 20% myeloid cells^18^. We first evaluated differences in the immune cells in the CSF. As shown in Fig. 1a, b, different ratios of myeloid cells and lymphocytes could be detected in the CSF. Compared with those in the CSF of healthy controls, the percentages of neutrophils and CD8 + T cells in the CSF of GBM patients were reduced (Fig. 1c, g). The percentages of monocytes/macrophages and DCs were significantly increased in the CSF of GBM and GII–III patients (Fig. 1d, e), while the proportion of CD4 + T cells was not significantly different between the GBM and GII–III patients (Fig. 1f). The subsequent immunophenotyping results showed that the CD8 + T cells in GBM-CSF were in an immunosuppressive state, exhibiting low clonal activity (Ki67 expression) (Fig. 1h) and Type II IFN response inactivation (IFN-γ expression) (Fig. 1i). Immunophenotyping analysis of DCs showed that the activity of DCs in GBM patients was much lower than that in GII–III patients and even lower than that in healthy subjects without tumor antigen stimulation; GBM DCs exhibited decreased expression of antigen-recognized HLA-A (Fig. 1j), the costimulatory molecules CD40 and CD86 (Fig. 1k, l) and the antigen-processing protein TAP1 (Fig. 1m). These data show that GBM-CSF may lack functional DCs for the presentation of tumor-associated antigens (TAAs), which may be the reason GBM is more malignant than other types.

### Exos isolated from the CSF of GBM patients inhibit the antigen-presenting activity of DCs

**Fig. 2:**
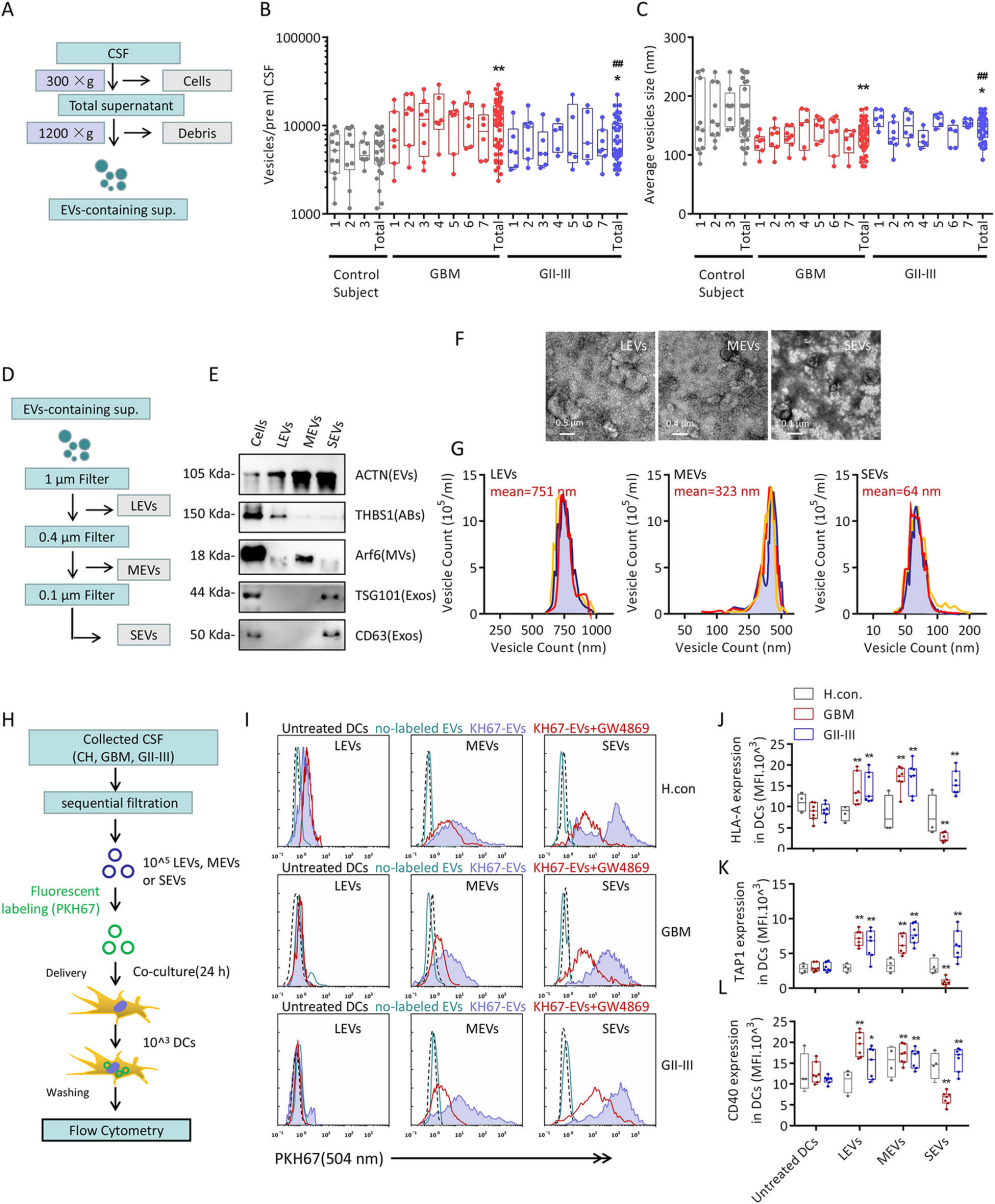
Extracellular vesicle analysis and dendritic cell uptake. **a** EV-containing supernatant from the CSF of healthy controls, GII–GIII patients or GBM patients. The CSF samples were cleared of cellular debris and dead cells by centrifugation. **b** and **c** Nanosight nanoparticle tracking analysis (NTA) of CSF-EVs. The number of vesicles and mode vesicle size for the indicated CSF sample are shown. Boxplots represent an average of seven NTA readings per CSF sample, and data were analyzed for statistical significance using a two-tailed Welch’s unequal variance’s *t*-test using healthy control data as the comparator. **d** Schematic of the vesicle isolation procedure involving sequential filtration. EV-containing supernatants were partitioned by filtering through filters with consecutively smaller pore sizes (1, 0.4, and 0.1 μm). **e** Representative graph of western blotted protein markers that specify different EV populations. pan-EVs (ACTN), apoptotic bodies (THBS1), microvesicles (ARF6), and exosomes (TSG101 and CD63). Each test was repeated at least twice. **f** Representative graph of negative staining transmission electron microscopy (TEM) of EVs collected by filter isolation. Scale bars: LEVs, 500 nm; SEVs, 400 nm; SEVs, 100 nm. Each test was repeated three times. **g** Count and size distribution of EVs collected by filter isolation. Each test was repeated at least three times. **h** Schematic of DC uptake of EVs collected by sequential filtration of GII–GIII or GBM patient CSF. **i** Quantification of the PKH67 fluorescence intensity of DCs that took up PKH67-labeled EVs from GII–GIII or GBM patient CSF. DCs were preincubated with GW4869, an EV uptake inhibitor, for 24 h. Each test was repeated three times. **j**–**l** Quantification of the MFIs of HLA-A, CD40, and TAP1 expression in DCs that took up EV populations from GII–GIII or GBM patient CSF. Each test was repeated five times. Data are shown as the mean ± SD; *p < 0.05 and **p < 0.01 by *t*-test.

A number of studies have shown that EVs serve as mediators of the tumor immune microenvironment, regulating tumor progression and maintenance^19,20^. EV-containing supernatant (Fig. 2a) was isolated from the CSF, the EVs were evaluated with nanoparticle tracking analysis (NTA) to observe the counts and particle sizes (Fig. 2, c), GBM and GII–III patients exhibited higher EVs concentrations than normal donors, and the average EVs particle size was also smaller in the GBM and GII–III patients than in normal donors. To separate EVs with different particle sizes, as shown in Fig. 2d, 0.1, 0.4, and 1-μm pore-size filters were used to separate CSF-derived EVs into large extracellular vesicles (LEVs; ≥0.4 μM; ≤1 μM), moderate extracellular vesicles (MEVs; ≥0.1 μM; ≤0.4 μM) and small extracellular vesicles (SEVs; ≤0.1 μM). LEVs, MEVs and SEVs have surface markers similar to those of apoptotic bodies (ABs) (THBS1), microvesicles (MVs) (Arf6) and Exos (TSG101 and CD63) (Fig. 1e). Negative staining transmission electron microscopy (TEM) also revealed that their sizes and morphologies were consistent with those reported for ABs, MVs, and Exos^21–23^(Fig. 1f, g). PKH67 was used to label EVs from healthy subjects, GBM patients and GII–III patients, and the EVs were cocultured with DCs in vitro (Fig. 1h). As shown in Fig. 1i, both MEVs and SEVs could be taken up by DCs, and the vesicle uptake inhibitor GW4869 could inhibit this process, while LEVs were hardly taken up by DCs. The effects of these EVs on the antigen-presenting activity of DCs were observed, as shown in Fig. 2j–l, and the SEVs/MEVs isolated from GII–III CSF and MEVs isolated from GBM-CSF could activate HLA-A, CD40 and TAP1 expression. The only special case was GBM-SEVs, which greatly reduced the antigen-presenting activity of DCs. Considering that Exos have the ability to carry tumor antigens, we detected TAAs in all EVs isolated from GII–III and GBM patients, but there was no obvious difference in the tumor antigens molecules carried by each type of EVs (Supplementary Fig. S1a). These results confirmed that Exos from GBM-CSF significantly inhibited the antigen-presenting activity of DCs.

### GBM-CSF-Exos contain specific protein cargo

**Fig. 3:**
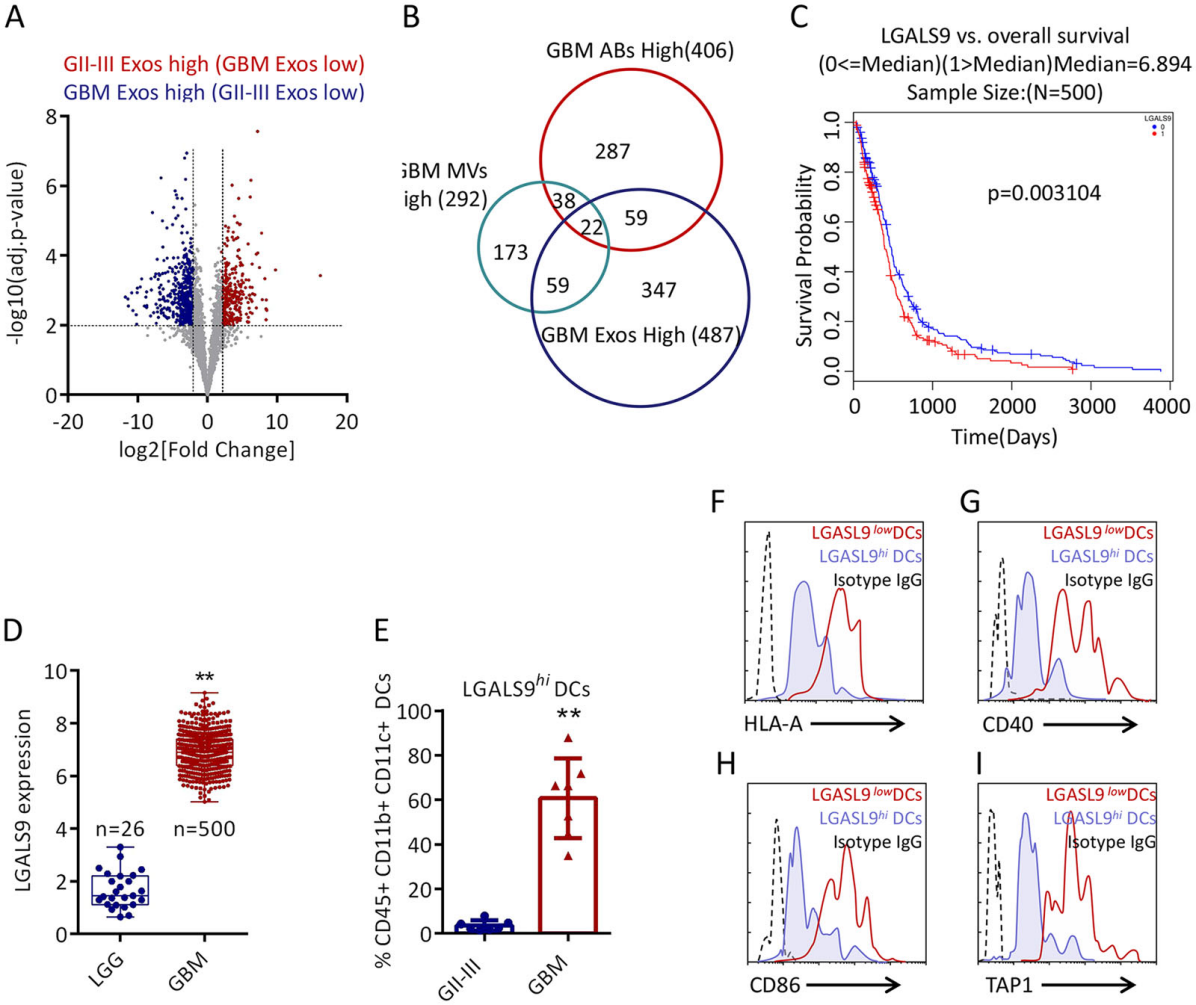
Exosomal LGALS9 is associated with GBM immunosuppression. **a** Volcano plot of significant proteins with a decreased (red; GBM-Exos-LOW) or increased abundance (blue; GII–III Exos LOW) in GBM relative to GII–III. **b** Venn diagram showing the overlap among GBM-ABs-High, GBM-MVs-High (292) and GBM-Exos-High (487). **c** Correlation between the expression of LGALS9 and the overall survival rate of GBM patients (*p* = 0.003). LGALS9 expression levels were dichotomized into high (red) and low (blue) values based on median population expression levels. The data were from LinkedOmics (http://www.linkedomics.org/). **d** Differences in the expression of LGALS9 in low-grade gliomas (GI, GII, and GIII, *n* = 5) and GBM (*n* = 500). The data were from LinkedOmics. **e** The difference in the proportion of LGALS9^*hi*^ DCs in the total CSF between GII–III (*n* = 7) and GBM (*n* = 7). **f**–**i** Representative flow cytometry plots of the differential expression of HLA-A, CD40, CD86, and TAP1 in LGALS9^hi^ DCs and LGALS9^low^ DCs in GBM-CSF. Data are shown as the mean ± SD; *p < 0.05 and **p < 0.01 by a *t*-test.

Quantitative tandem mass spectrometry (TMT) of cell pellets, ABs, MVs, and Exos isolated from healthy subject, GBM patient and GII–III patient CSF. As shown in Supplementary Table S1 and Supplementary Figs. S2a, b, most of the protein levels in the cell pellets of GBM-CSF and GII–III CSF samples were higher than those in EVs. Among the 2958 detectable proteins, 1569 proteins in the EVs were always higher than those in the cells (Supplementary Fig. S2c and S2h). In addition, pairwise Pearson’s correlation analysis showed that the protein content of each fraction was more similar by the type of vesicle rather than by the tumor type (Supplementary Fig. S2i), which indicates that particle size determines the heterogeneity of CSF-EVs. In GBM, 406 (ABs), 292 (MVs) and 487 (Exos) proteins exhibited higher expression than that in GII–GIII, as shown in Fig. 3a and Supplementary Fig. S3a, b. Considering that only Exos isolated from GBM-CSF have obvious DC inhibitory activity, Venn diagram analysis showed that there were 347 unique protein cargos in GBM-CSF-Exos (Fig. 3b). Gene ontology (GO) analysis and Kyoto Encyclopedia of Genes and Genomes (KEGG) pathway analysis showed that these GBM-CSF-Exos unique protein cargos are involved in various cellular processes, such as antigen presentation, cell adhesion and cell metabolism (Supplementary Fig. S3c–e). There are 12 + 6 proteins related to endogenous antigen presentation, as shown in Supplementary Fig. S3. Among this cargo proteins, LGALS9 attracted our attention. The interaction between LGALS9 and TIM3 is found in most tumors, and TIM3 is a well-known important immune checkpoint^24–26^. However, no studies have focus on whether LGALS9 in GBM exosomes can induce inhibitory immunity in the tumor microenvironment, so we focused our attention on LGALS9. In data from The Cancer Genome Atlas (TCGA) database, high expression of LGALS9 in GBM patients (*n* = 500) represented worse survival (Fig. 3c). In contrast, the expression of LGALS9 was lower in low-grade glioma (LGG) (Fig. 3d). Although there are studies showing the correlation between LGALS9 and GBM, there is no research addressing whether LGALS9 is involved in the regulation of DCs. As expected, more LGALS9^*hi*^ DCs were detected in GBM-CSF than in GII–III CSF (Fig. 3e). These LGALS9^*hi*^ DCs had lower expression of CD40, CD86, HLA-A and TAP1 than LGALS9^*low*^ DCs (Fig. 3f–i). Therefore, LGALS9 exists uniquely in GBM-CSF-Exos, which may be related to presentation by active DCs.

### Exos derived from GBM cells inhibit the antigen-presenting activity of DCs

**Fig. 4:**
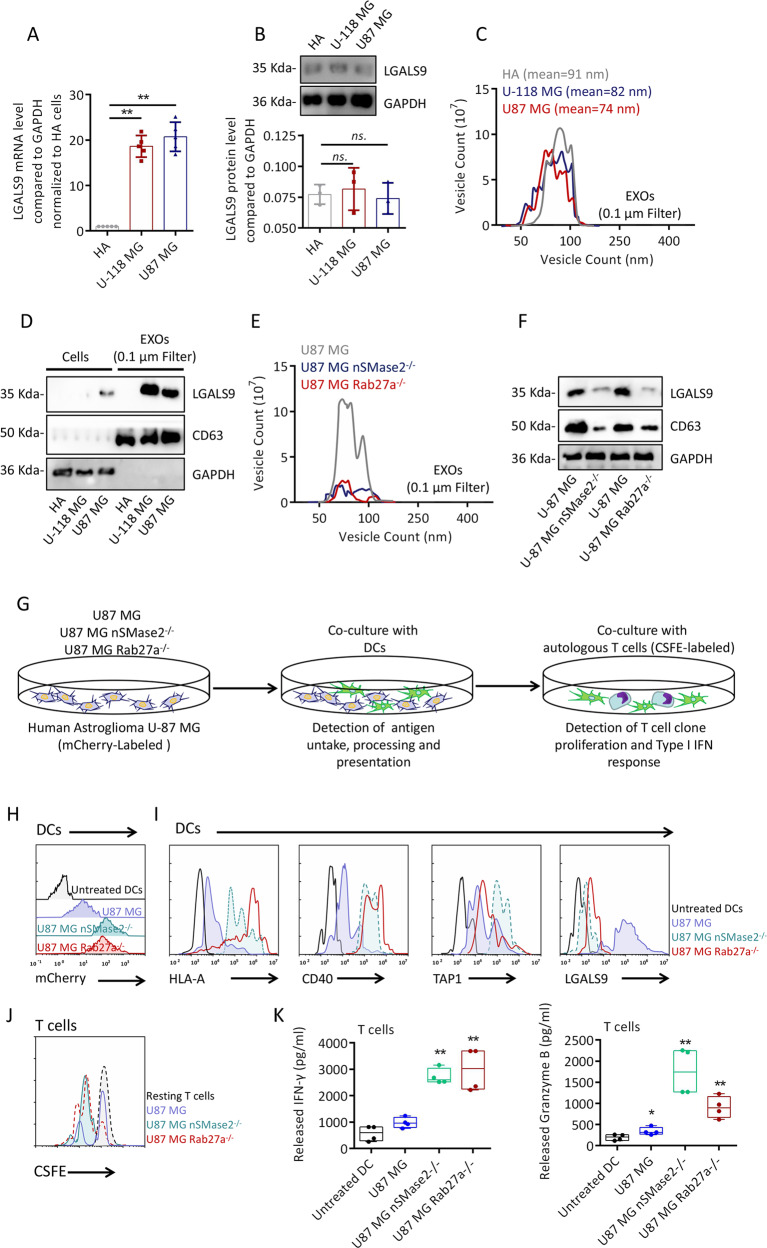
The effect of GBM-derived exosomes on antigen presentation by DCs. **a** qRT-PCR showing the difference in the transcription of LGALS9 in U118 MG and U87 MG cells compared with HA cells. GAPDH was used as an internal reference. **b** Western blot analysis showing differences in the protein level of LGALS9 in U118 MG and U87 MG cells. **c** NTA showing the differences in particle size and number of U118 MG^-^ and U87 MG-Exos passed through a 0.1-μM filter. **d** Western blot analysis of Exos secreted by U118 MG and U87 MG cells. The content of LGALS9 was different from that of HA cells. **e** NTA showing the effect of CRISPR/Cas9 knockout of *Rab27a* and *nSMase2* in U87 MG cells on the sizes and counts of secreted Exos compared with U87 MG WT Exos. **f** Western blot analysis comparing the U87 MG Rab27a^−/−^ and U87 MG nSMase2^−/−^ cell levels of the secreted exosome markers TSG101 and CD63 with those of U87 MG WT cells in the same volume of cell culture medium. **g** Schematic diagram of coculture experiments evaluating DC antigen presentation to U87 MG cells and activation of T cells. **h** Representative flow cytometry plots of the mCherry fluorescence intensity in U87 MG WT, U87 MG Rab27a^−/−^ and U87 MG nSMase2^−/−^ DCs. **i** Representative graph of HLA-A, CD40, TAP1, and LGALS9 expression in U87 MG WT, U87 MG Rab27a^−/−^ and U87 MG nSMase2^−/−^ DCs. **j** Flow cytometry analysis of the fluorescence intensity of CFSE in T cells exposed to DCs presenting different U87 MG antigens. **k**, **l** ELISA to detect the release of IFN-γ and granzyme B into cell culture medium by T cells in contact with DCs presenting designated U87 MG antigens. All data are shown as the mean ± SD; *p < 0.05 and **p < 0.01 by a *t*-test, and each test was repeated three times.

Owing to the complex cell environment of the CSF, the source of GBM-CSF-Exos that highly express LGALS9 is unknown. We attempted to compare the levels of LGALS9 transcription and translation between malignant glioblastoma lines (U118 MG and U87 MG) and primary astrocytes (HA). The transcriptional level of LGALS9 in U118 MG and U87 MG cells was nearly 20 times higher than that in HA cells (Fig. 4a); however, the translation level of LGALS9 was not significantly different among the cells (Fig. 4b). To rule out an inconsistency in the translation rate, leading to differences in LGALS9 protein levels, as shown in Supplementary Fig. S4a, b, polysome fractionation analysis experiments showed that U118 MG and U87 MG cells had the same translation efficiency as HA cells. To rule out the effect of the LGALS9 protein degradation rate, the small molecules bafilomycin A1 (BafA1)^27^ and MG132^28^ were used to interfere with lysosome and proteasome activities, respectively. As shown in Supplementary Fig. S4c, d, the difference in LGALS9 protein degradation could not explain the differences in the mRNA and protein levels. Therefore, we considered the possibility of differential secretion of LGALS9 from cells in the form of membrane-bound vesicles. Separating HA-, U118 MG^-^, and U87 MG-Exos with an ultrafiltration membrane, as shown in Fig. 4c, showed that the U118 MG^-^ and U87 MG-Exos had a slightly smaller particle size than the HA-Exos, while the content of LGALS9 was nearly a hundred-fold higher in the U118 MG^-^ and U87 MG-Exos than in the HA-Exos (Fig. 4d). Therefore, even if the LGALS9 protein level is low at the cellular level, U118 MG and U87 MG cells package more LGALS9 into Exos. To observe whether these Exos affect the activity of DCs, CRISPR/Cas9 was used to knock out Rab27a and nSMase2, two components that affect the assembly and release of Exos^29^, in U87 MG cells. As shown in Fig. 4e, f, U87 MG nSMase2^−/−^ and U87 MG Rab27a^−/−^ cell Exo secretion was significantly reduced. As shown in Fig. 4g, we designed a coculture experiment to observe the activation of T cells by tumor antigen-activated DCs. The uptake of fluorescent mCherry-positive U87 MG cells by DCs was used to indirectly observe the uptake of tumor antigens. The mCherry uptake of DCs was significantly enhanced by coculture with U87 MG nSMase2^−/−^ and U87 MG Rab27a^−/−^ cells (Fig. 4h). The expression of CD40, HLA-A and TAP1 in DCs activated by U87 MG nSMase2^−/−^ and U87 MG Rab27a^−/−^ cells was higher than that of U87 MG WT cells, while the expression of LGALS9 in DCs was lower than that in U87 MG WT cells (Fig. 4i). Active DCs will activate the clonal proliferation (carboxyfluorescein succinimidyl ester (CFSE) incorporation) and type I IFN response (IFN-γ and Granzyme B release) of cocultured T cells, and these processes were significantly enhanced in the U87 MG nSMase2^−/−^ and U87 MG Rab27a^−/−^ coculture system (Fig. 4j–l). These data indicate that Exos derived from GBM inhibit DC antigen presentation and cytotoxic T-cell immunity.

### Exosomal LGALS9 inhibits the activation of DCs and promotes tumor progression

**Fig. 5:**
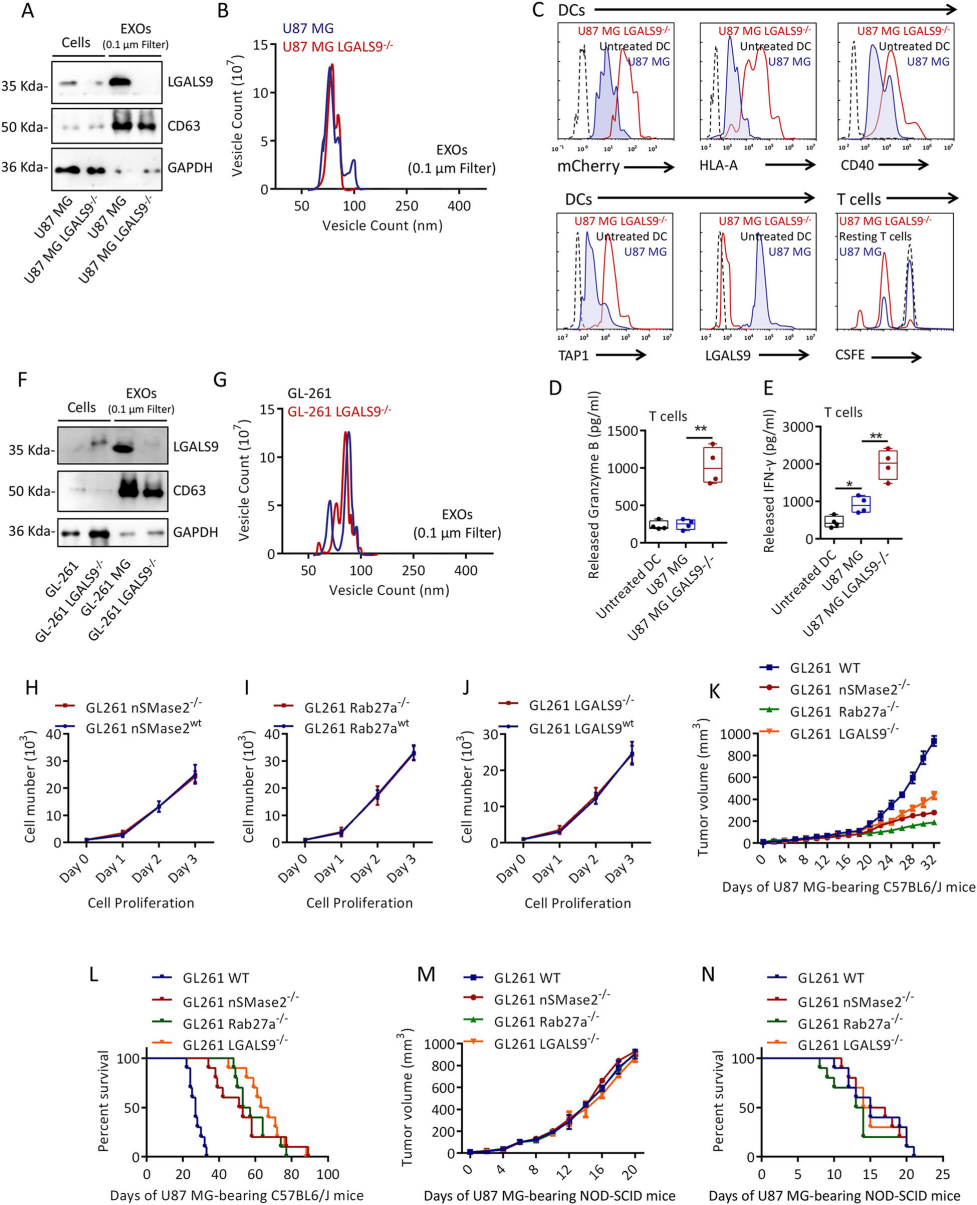
The effect of exosomal LGALS9 on the antigen presentation of DCs. **a** Western blot analysis of the expression of LGALS9 and CD63 in U87 MG LGALS9^−/−^ cells and exosomes. **b** NTA of U87 MG LGALS9^−/−^ and U87 MG WT Exos. **c** Flow cytometry detection of mCherry, HLA-A, CD40, TAP1 and LGALS9 in DCs exposed to designated U87 MG antigens and CFSE in T cells exposed to active DCs. **d**, **e** ELISA detection of the release of IFN-γ and granzyme B into cell culture medium by T cells exposed to DCs presenting designated U87 MG antigens. **f** The expression of LGALS9 and CD63 in GL-261 LGALS9^−/−^ cells and exosomes. **g** NTA of the differences in particle sizes and counts in equal volumes of the cell culture medium of GL-261 LGALS9^−/−^ and GL-261 wt cells. **h**–**j** CCK-8 detection of the proliferative activity of designated GL-261 cells. Tumor growth curves (**k**) and survival curves (**l**) of designated GL-261 tumor-bearing C57BL/6J mice. Tumor growth curves (**m**) and survival curves (**n**) of designated GL-261 tumor-bearing immunodeficient NOD-SCID mice. All data are shown as the mean ± SD; *p < 0.05 and **p < 0.01 by a *t*-test, each cell test was repeated three times, and the tumor-bearing mouse experiment were set up with 14 mice per group.

CRISPR/Cas9 was used to knock out LGALS9 in U87 MG cells (Fig. 5a), but this did not affect the amount of U87 MG-Exos released (Fig. 5b). The ability of U87 MG LGALS9^−/−^ antigens to activate DCs and T cells was higher than that of U87 MG WT antigens (Fig. 5c–e). These data further clarify the immunosuppression of DCs by exosomal LGALS9 derived from GBM cells. In addition, the LGALS9 receptor TIM3 was knocked out in cocultured DCs. As shown in Supplementary Fig. S5a, the level of TIM3^−/−^ DCs activated by coculture with U87 MG cells was higher than that of WT DCs (Supplementary Fig. S5b, c). This indicates that the inhibition of DCs by exosomal LGALS9 is TIM3 dependent. To observe whether LGALS9 affects tumor progression in vivo, mouse GL-261 GBM cells were used to construct a tumor-bearing mouse model. LGALS9-knockout GL-261 cells had the same effect on Exos as human U87 MG cells (Fig. 5f, g). The proliferative activity of GL-261 cells in which nSMase2, Rab27a, and LGALS9 were knocked out was not significantly inhibited (Fig. 5h–j). GL-261 cells with a WT, nSMase2^−/−^, Rab27a^−/−^, or LGALS9^−/−^ genotype were injected subcutaneously into the abdominal region of syngeneic C57BL6/J mice, which were then observed for >3 months. In mice implanted with GL-261WT cells for ~20 days, tumor growth was observed, and the longest survival period was 32 days (mice euthanized after exceeding the upper limit of tumor growth) (Fig. 5k). In contrast, the growth rate of subcutaneous tumors formed from GL-261nSMase2^−/−^, Rab27a^−/−^, or LGALS9^−/−^ cells was much slower, and the survival times associated with these cells were also significantly longer than that of GL-261 WT tumor-bearing mice (Fig. 5l). In addition, we used NOD-SCID mice (deficient in T cells, B cells and DCs) instead of C57BL6/J mice as tumor-bearing hosts. In stark contrast to those of normal mice, in the background of immunodeficiency, the tumor growth rate and survival time of GL-261nSMase2^−/−^, Rab27a^−/−^, and LGALS9^−/−^ tumor-bearing mice were not significantly different from those of GL-261WT tumor-bearing mice. (Fig. 5m, n). Therefore, exosomal LGALS9 promotes tumor growth by suppressing the immune system.

### Exosomal LGALS9 participates in immunosuppression in mouse CSF

**Fig. 6:**
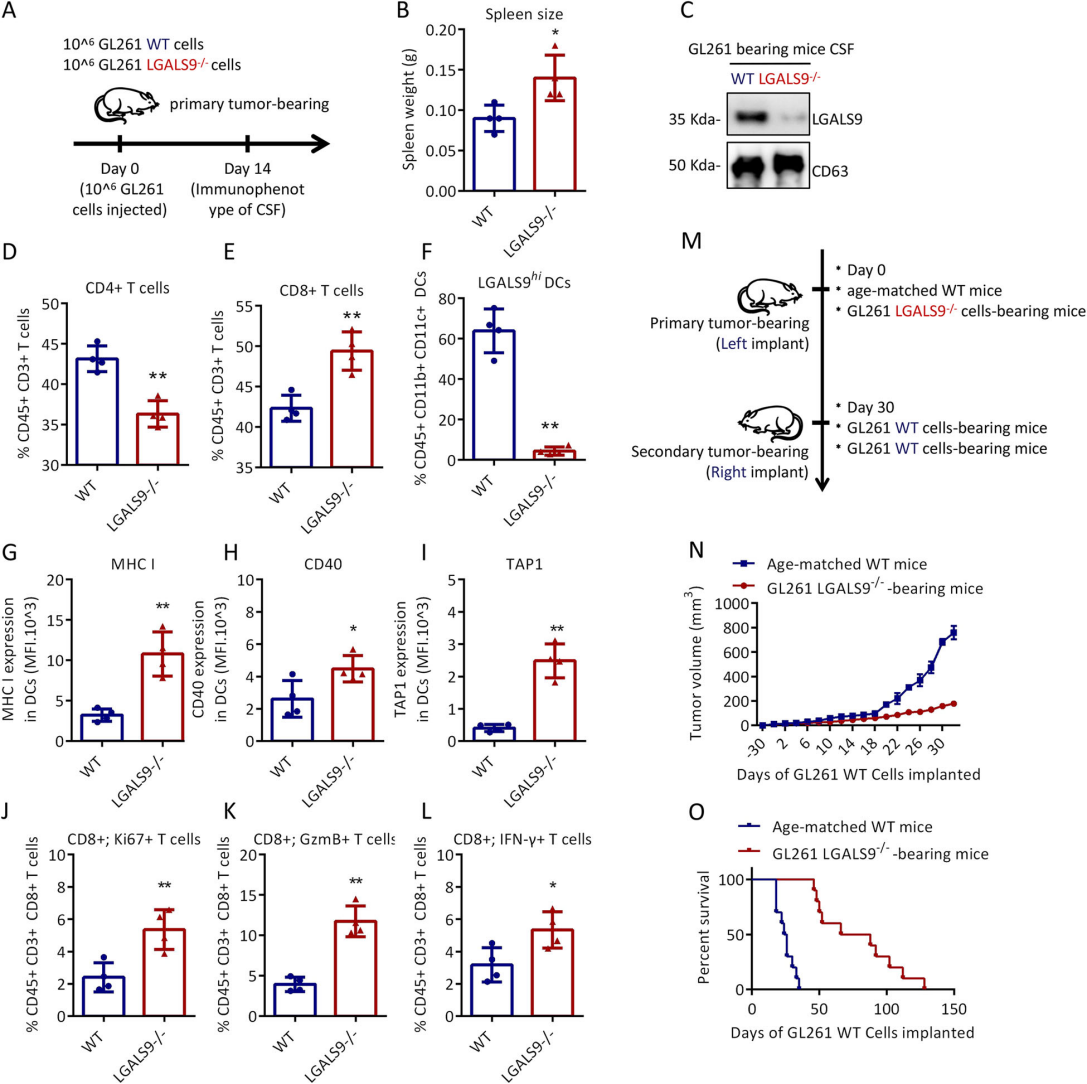
Effect of exosomal LGALS9 on the mouse CSF immune microenvironment. **a** Schematic of the experimental design for (**b**–**i**). Briefly, four mice were transplanted with GL-261 LGALS9^−/−^ cells or GL-261 WT cells, followed by immune analysis at 14 days. Spleen size (**b**) and LGALS9 expression in the CSF (**c**) of GL-261 LGALS9^−/−^ and GL-261 WT tumor-bearing mice. The percentages of LGALS9^*hi*^ DCs (**d**), CD8 + T cells (**e**), and CD4 + T cells (**f**) in the CSF of tumor-bearing mice. The expression of MHC I (**g**), CD40 (**h**), and TAP1 (**i**) in DCs in the CSF. The percentage of KI67 + cells among CD8 + T cells (**j**), the percentage of Gzm B + cells (**k**) and the percentage of IFN-γ-positive cells (**l**) in the CSF. **m** Schematic of the experimental design for **n**–**o**. Fourteen mice were transplanted with GL-261 LGALS9^−/−^ cells or GL-261 WT cells, followed by secondary contralateral subcutaneous injection of GL-261 WT cells on day 30. The tumor growth volume (**n**) and survival rate (**o**) in the following days. All data are shown as the mean ± SD; *p < 0.05 and **p < 0.01 by a *t*-test.

Next, we verified whether the loss of exosomal LGALS9 had a similar effect on the antitumor immune response. To this end, we determined the numbers of myeloid cells and lymphocytes in GL-261 WT and GL-261 LGALS9^−/−^ primary brain tumor-bearing mouse CSF (Fig. 6a). On day 14, the spleen of GL-261 LGALS9^−/−^ tumor-bearing mice was significantly larger than that of GL-261 WT tumor-bearing mice (Fig. 6b). The expression of LGALS9 was almost undetectable in the CSF of GL-261 LGALS9^−/−^ tumor-bearing mice (Fig. 6c). The ratio of CD4 + T cells to CD8 + T cells was significantly changed in the GL-261 LGALS9^−/−^ tumor-bearing mouse CSF (Fig. 6d, e). In the CSF of GL-261 WT tumor-bearing mice, more than 60% of DCs highly expressed LGALS9, while LGALS9^*hi*^ DCs in GL-261 LGALS9^−/−^ tumor-bearing mice accounted for less than 5% of the total DC population (Fig. 6f). More importantly, the expression of MHC I, CD40 and TAP1 in DCs in GL-261 LGALS9^−/−^ tumor-bearing mouse CSF was significantly higher than that in DCs in GL-261 WT tumor-bearing mouse CSF (Fig. 6g–i). As expected, compared to that of GL-261 WT tumor-bearing mouse CD8 + T cells, the antitumor immunity of GL-261 LGALS9^−/−^ tumor-bearing mouse CD8 + T cells was enhanced, with an increased proportion of Ki67-positive cells and enhanced expression of IFN-γ/GzmB (Fig. 6j–l). These data show that the reductions in exosomal LGALS9 expression and LGALS9^*hi*^ DC accumulation restored the antitumor activity of the CSF microenvironment of GBM. Since GBM inhibits the antigen presentation of DCs and the activation of CD8 + T cells through exosomal LGALS9, we propose the hypothesis that when Exos lack LGALS9, immunocompetent mice should be able to not only directly inhibit LGALS9-deficient GL-261 cells but also produce immune memory specific for GL-261 cells.

To test this hypothesis, GL-261 LGALS9^−/−^ cells were implanted subcutaneously in one side of C57BL6/J mice. After 30 days, GL-261 WT cells were implanted subcutaneously in the other side (Fig. 6m). Interestingly, the initial exposure to GL-261 LGALS9^−/−^ cells led to resistance to challenge with GL-261 WT cells, resulting in tumor growth inhibition and improved survival rates (Fig. 6n, o). Therefore, this finding shows that once DCs are exposed to antigens when exosomal LGALS9 is not present, after completing the entire antigen presentation process, they will become resistant to the immunosuppressive effect of exosomal LGALS9.

## Discussion

Owing to its high invasiveness, heterogeneity and recurrence rate and limited treatment options, GBM is almost always fatal within a few years. In contrast, LGG is weakly invasive and usually characterized by IDH mutations, resulting in a better prognosis^30^. Body fluid biopsy is essential for the diagnosis, progression monitoring and prognostic analysis of a tumor, but the difficulty of brain biopsy makes CSF sampling have obvious advantages in material collection^31^. Exos have attracted much attention in the diagnosis and treatment of GBM^32^. Several studies have shown that CSF Exo-encapsulated miRNAs can be used to appropriately assess the potential molecular genetic status, drug resistance and prognosis of GBM^33–35^. The correlation between CSF-Exo-contained proteins and the progression of GBM is not clear. Before evaluating EVs, we first tested the immune cell composition of the CSF. The proportions of immune cells in the CSF of GII–III and GBM were significantly different. There were more myeloid cells and lymphocytes in the CSF of GII–III patients, with more CD8 + T cells with antitumor activity and DCs with antigen-presenting activity. In contrast, although the proportion of DCs in the CSF of GBM was also relatively increased, most of these cells were ineffective and insufficient for tumor antigen presentation. Strong immunosuppression is the main feature of GBM and is exacerbated by the background effect of the chemotherapeutic drug temozolomide (TMZ)^8^. Therapies that reverse GBM immunosuppression, such as oncolytic viral therapy^36^, vaccination therapy^37^, chimeric antigen receptor (CAR) T-cell therapy^38^, and immune checkpoint blockade^39^, which were all designed to improve the immunosuppressive status of GBM, are feasible. As Exos in the CSF directly serve as communication carriers for these immune cells and cancer cells, it is reasonable to conclude that Exos have immunomodulatory effects and speculate on potential therapeutic targets.

As a broad term, EVs include at least three main subgroups^40,41^: ABs (400–1000 nm) produced by apoptosis, MVs (100–400 nm) formed by exocytosis, and Exos secreted by endosomal compartments (40–100 nm). The cargo of EVs is composed of proteins, nucleic acids and other biological molecules. These cargo molecules are based on the identity, molecular state and biological activity of the originating cell. Initially, EVs were thought to have antitumor effects because they carried tumor antigens^42^. Subsequent studies have shown that EVs have direct immune cell-suppressing effects, such as effects on DCs^43^, NK cells^44^ and CD8 + T cells^45^, that accelerate tumor progression. This controversy stems from the heterogeneity of EVs, that is, the origin of EVs is complex, they carry a variety of goods, and target cells take up EVs via different mechanisms. Our data also showed that the protein content of each EV fraction was more similar when results were stratified by the type of vesicle than when they were stratified by tumor type. In fact, we detected TAAs in all grouped EVs from GII–III and GBM patients, and there was no obvious difference in the tumor components carried by the EVs. GII–III-CSF-Exos/MVs and GBM-CSF MVs could be taken up by DCs and activate DCs. The only special case was GBM-Exos, which strongly reduced the antigen-presenting activity of DCs.

Compared with GII–III-Exos, GBM-Exos contained 347 unique proteins (GBM-Exos-high) that were not present in GBM-MVs or GBM-Abs. GBM-Exo-high proteins are involved in metabolism (such as the oxidation-reduction process, mitochondrial respiratory chain complex I assembly and glutathione metabolic process, but they may indicate that EVs in GBM specimens are contaminated by cells or reflect the high-energy status of GBM tumors) and the biological process of antigen presentation (antigen processing and presentation of exogenous peptide antigen via MHC class II or via MHC class I in a TAP-dependent manner). Among the cargo molecules, LGALS9 attracted our attention. LGALS9 (also known as Gal-9) has been proven to be associated with cancer, especially in hematological tumors^24^. LGALS9 is a sign of a poor prognosis for all grades of glioma^29^. In fact, the expression of LGALS9 in GII–III is lower than that in high-grade GBM. It is worth noting that LGALS9 is a ligand of TIM3 and that the LGALS9/TIM3 pathway has immunoregulatory effects such as regulating T helper cell apoptosis^25,26^, exhausting CD8 + T cells^46^ and activating CD4 + T cells^47^ and NK cells^48^. However, the origin and target cells of these GBM-Exos-LGALS9 are still unknown.

However, it is difficult to explore the cell origin of Exos-LGALS9 in GBM-CSF clinical samples. Therefore, we observed the activity of exosomal LGALS9 released by GBM cells in vitro. After excluding differences in transcription efficiency and protein degradation efficiency, we demonstrated that U118 MG and U87 MG cells continually assembled LGALS9 into Exos. U87 MG-Exos or exosomal LGALS9 could directly block the recognition, uptake, processing and presentation of U87 MG antigens by DCs and inhibit the expression of costimulatory molecules and clonal proliferation of cocultured T cells and the type I IFN antitumor response. In addition, TIM3 deficiency in DCs could reverse the inhibition of DCs by exosomal LGALS9, which indicates that exosomal LGALS9 is TIM3 dependent. In a tumor-bearing mouse model, it was difficult for LGALS9-deficient GBM cells to grow in the presence of a healthy immune system, but in immunodeficient mice, this phenomenon was eliminated. Immunocompetent mice completed the entire antigen presentation process and cytotoxic T lymphocyte killing process. Further experiments showed that mice immunized with GL261 LGALS9^−/−^ tumors still had sustained anticancer immune memory when they were inoculated with GL261 WT cells, which ultimately inhibited tumor growth and prolonged survival in the mice. In conclusion, we reveal that GBM cell-derived exosomal LGALS9 acts as a major regulator of tumor progression by inhibiting DC antigen presentation and cytotoxic T-cell activation in the CSF and that loss of this inhibitory effect can lead to durable systemic antitumor immunity.

## Supplementary information

Supplement Figure and Table Legends

SUPPLEMENTAL Figure 1

SUPPLEMENTAL Figure 2

SUPPLEMENTAL Figure 3

SUPPLEMENTAL Figure 4

SUPPLEMENTAL Figure 5

SUPPLEMENTAL TABLE 1

SUPPLEMENTAL TABLE 2

SUPPLEMENTAL TABLE 3
