# Supplementary material for: Exosomal LGALS9 in the cerebrospinal fluid of glioblastoma patients suppressed dendritic cell antigen presentation and cytotoxic T-cell immunity
Source: Cell Death Dis. 2020 Oct 22;11(10):896. doi: 10.1038/s41419-020-03042-3 (PMC7582167; doi:10.1038/s41419-020-03042-3)
Supplement: Supplementary file 1 — Supplement Figure and Table Legends [file 41419_2020_3042_MOESM1_ESM.doc]

**Supplementary figure and table legends**

**Figure S1. The content of tumor-associated antigens in extracellular vesicle fractions.**

Representative Western blot analysis of specific TAA (GII-GIII (mlDH1), GBM (EGFR)) and nonspecific TAA (MAGEA1 and HER2) expression in LEVs, MEVs and SEVs in H. con, GII-III and GBM CSF. ACTN is a pan-EV marker, THB1 is an AB-specific marker, ARF6 is an MV-specific marker, and TGS101 is an Exo-specific marker.

**Figure S2. The composition of protein cargos in extracellular vesicle fractions and correlations with the type of EV.**

Unsupervised hierarchical clustering analysis of the z-score proportional protein expression values of all cargo proteins identified in GBM (A) and GII-III (B) CSF cell, LEV, MEV and SEV protein fractions. Volcano plot of significant proteins with a decreased or increased abundance in LEVs (C), MEVs (E) or SEVs (G) relative to the abundance in GBM cells. Volcano plot of significant proteins with a decreased or increased abundance in LEVs (D), MEVs (F) or SEVs (H) relative to the abundance in GII-III cells.(I) Pearson correlation coefficients of a pairwise analysis of the expression levels of cell and EV fractions from GII-III and GBM CSF. The values of the coefficient are indicated and marked with background colors (> 0: significant trend; < 0: suggested trend).

**Figure S3. Quantitative proteomics of filtered EV fractions.**

(A) Volcano plot of significant proteins with a decreased (red; GBM‐LEV-LOW) or increased abundance (blue; GII-III‐LOW) in GBM LEVs relative to the abundance in GII-III LEVs. (B) Volcano plot of significant proteins with a decreased (red; GBM‐LEV-LOW) or increased abundance (blue; GII-III‐LOW) in GBM LEVs relative to that in GII-III LEVs. (C-F) The biological process, cellular component, molecular function and KEGG pathway enrichment of unique GBM CSF SEV protein cargo determined using the DAVID database for functional annotation and enrichment analysis.

**Figure S4. Differences in the translation rates and protein degradation of LGALS9.**

(A) Polysome profiles in HA, U87 MG and U118 MG cells. (B) Composition of LGALS9 mRNA across the monosome, low-polysome, and heavy-polysome fractions in HA, U87 MG and U118 MG cells. (C) Representative protein expression of LGALS9 in HA and U87 MG cells in the presence or absence of the lysosome inhibitor BafA. LC3B was used as a positive control. (D) Representative Western blotting for LGALS9 in HA and U87 cells in the presence or absence of the proteasome inhibitor MG132. Ubiquitin was used as a positive control. Data are shown as the mean ± SD; *p < 0.05 and ** p < 0.01 by a t-test.

**Figure S5. Inhibition of DC antigen presentation by GBM exosomes is TIM3 dependent**

(A)Schematic diagram of coculture experiments for DC antigen presentation to U87 MG cells and activation of T cells. (B) Representative flow cytometry plots of the mCherry fluorescence intensity in TIM3-/- DCs or TIM3-WT DCs exposed to U87 MG antigens. (C) Representative graph of HLA-A, CD40, TAP1 and LGALS9 expression in TIM3^-/-^ DCs or TIM3-WT DCs exposed to U87 MG antigens. (D) The fluorescence intensity of CFSE in T cells exposed to TIM3^-/-^ DCs or TIM3-WT DCs presenting U87 MG antigens. (E) The release of IFN-γ and granzyme B into cell culture medium by T cells that contacted TIM3^-/-^ DCs or TIM3-WT DCs presenting U87 MG antigens. All data are shown as the mean ± SD; * p <0.05 and ** p <0.01 by a t-test, and each test was repeated 3 times.

**Table.s1.** Proteomics datas of EVs tested by Liquid chromatography and tandem mass spectrometry

**Table.s2.** Detailed characteristics of all patients.

**Table.s3.** sgRNA oligonucleotides
