# Supplementary figures and images for: Exosomal LGALS9 in the cerebrospinal fluid of glioblastoma patients suppressed dendritic cell antigen presentation and cytotoxic T-cell immunity
Source: Cell Death Dis. 2020 Oct 22;11(10):896. doi: 10.1038/s41419-020-03042-3 (PMC7582167; doi:10.1038/s41419-020-03042-3)

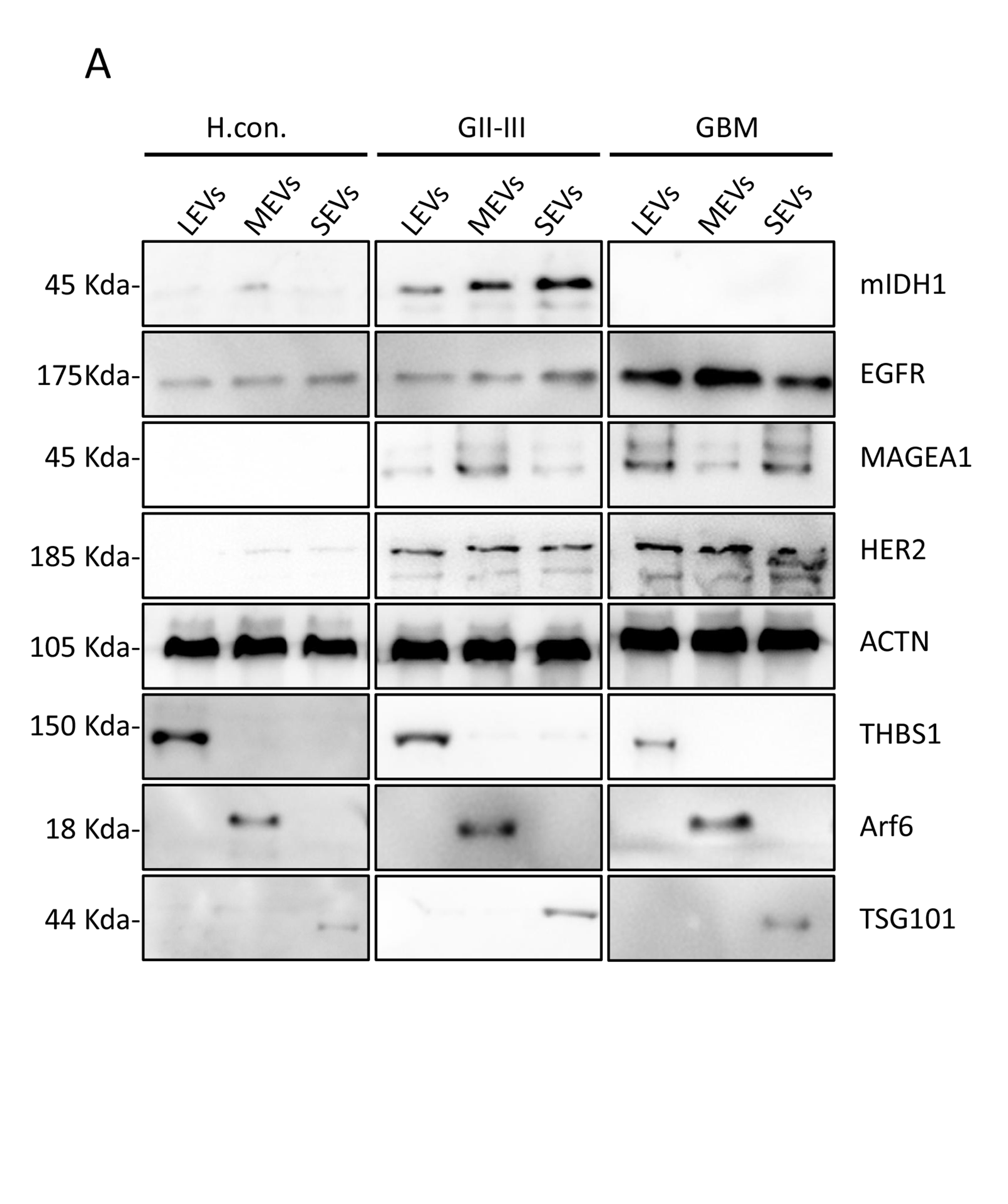

Supplement: Supplementary file 2 — SUPPLEMENTAL Figure 1 [file 41419_2020_3042_MOESM2_ESM.tif]

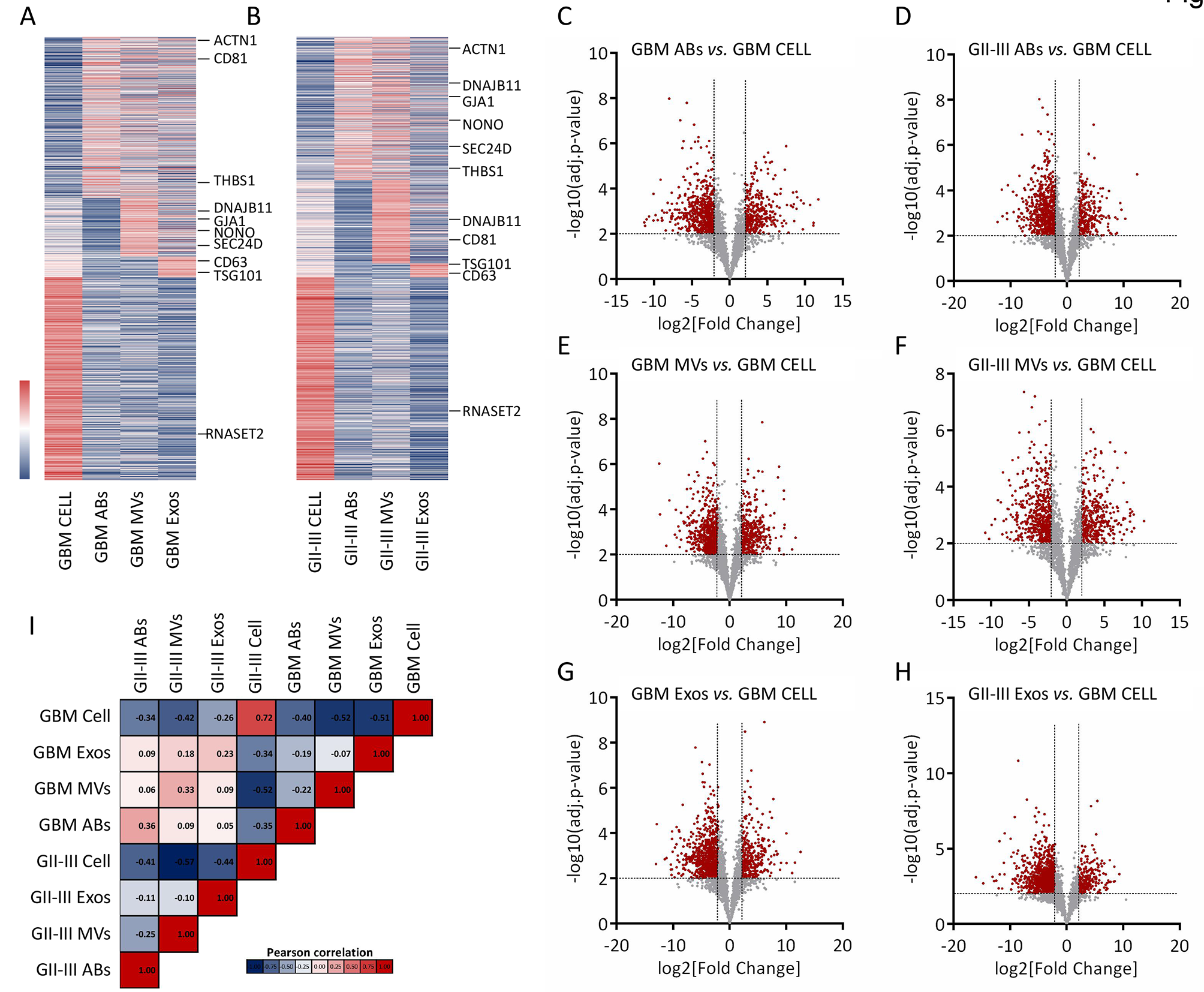

Supplement: Supplementary file 3 — SUPPLEMENTAL Figure 2 [file 41419_2020_3042_MOESM3_ESM.tif]

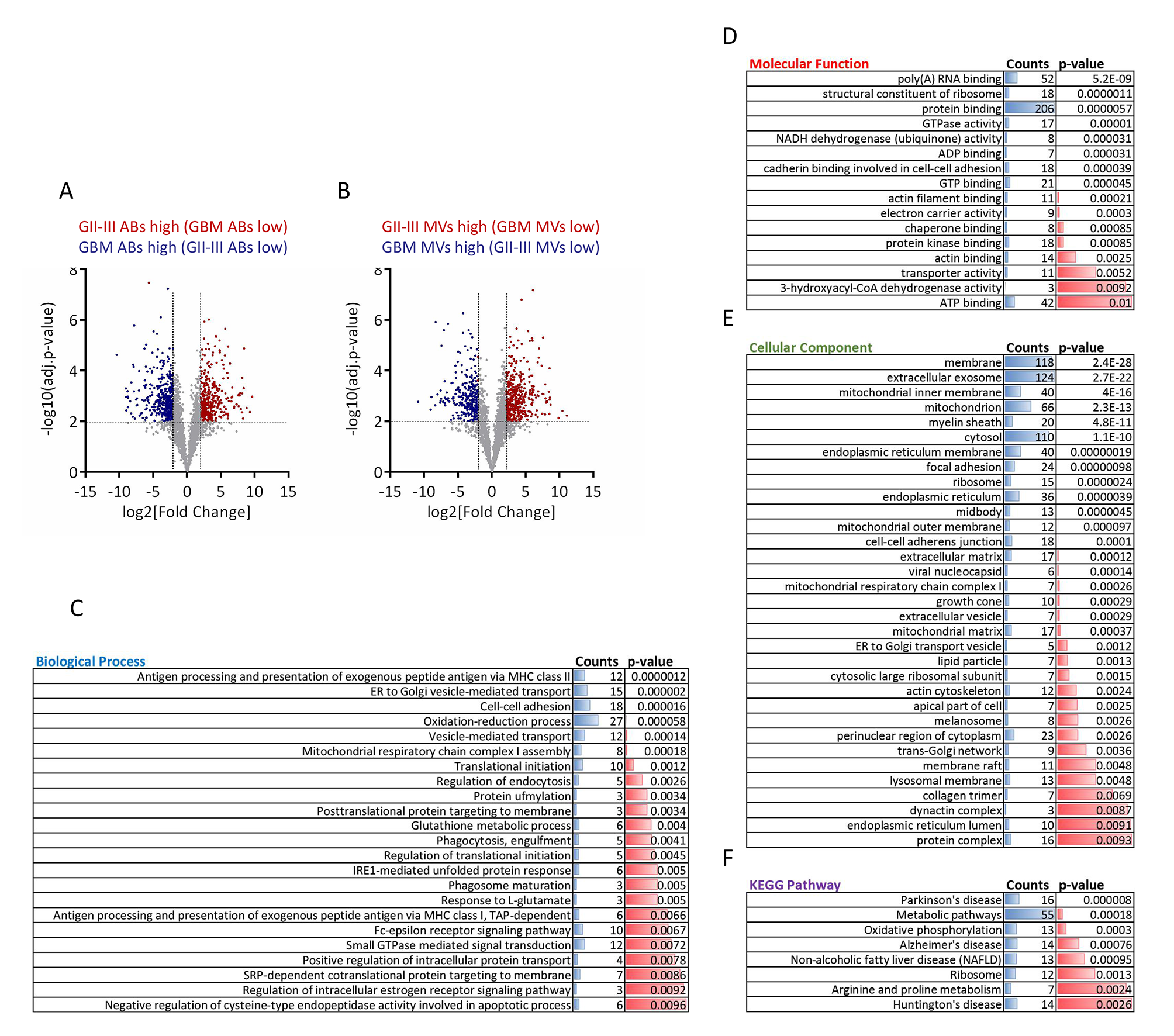

Supplement: Supplementary file 4 — SUPPLEMENTAL Figure 3 [file 41419_2020_3042_MOESM4_ESM.tif]

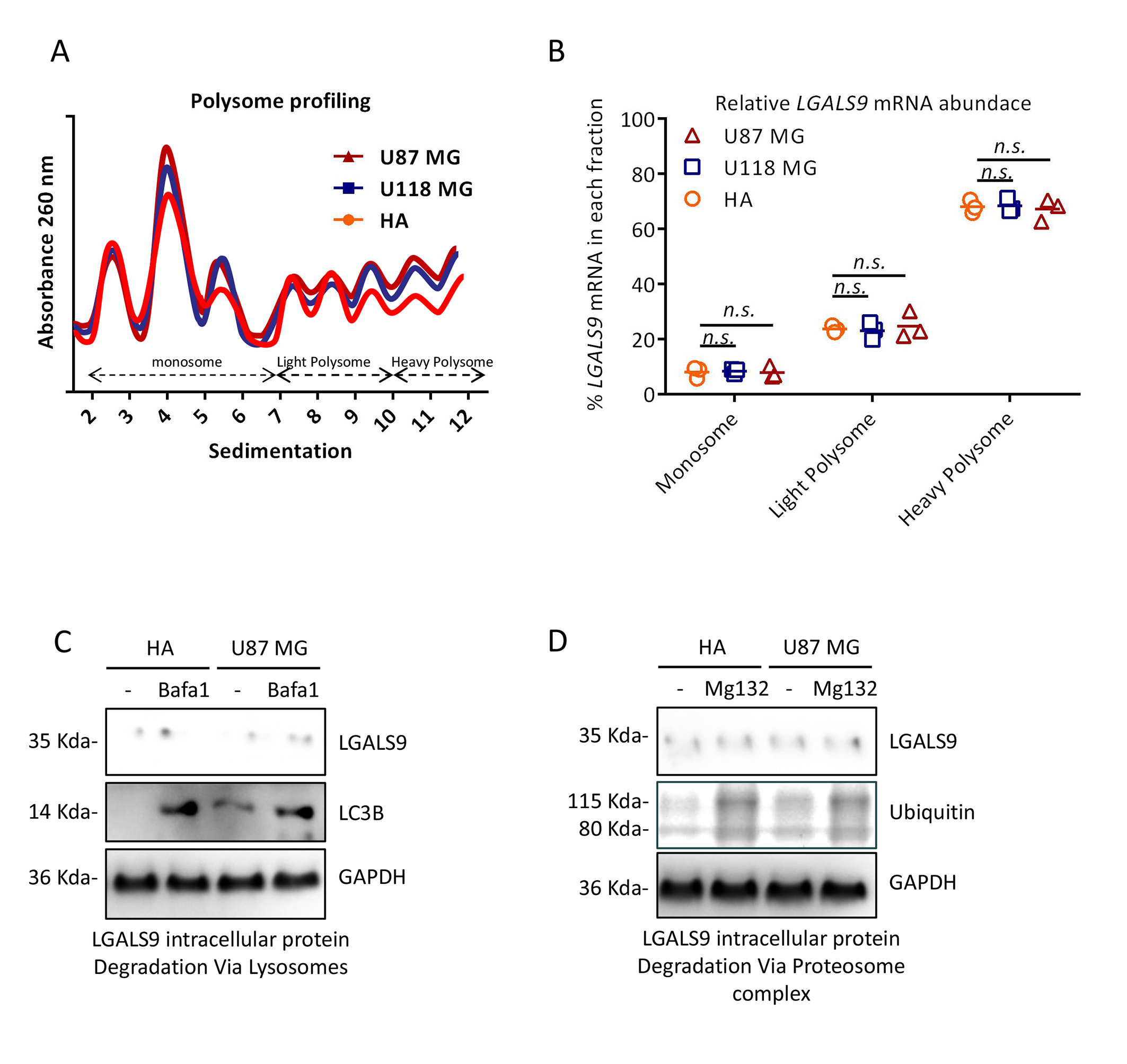

Supplement: Supplementary file 5 — SUPPLEMENTAL Figure 4 [file 41419_2020_3042_MOESM5_ESM.tif]

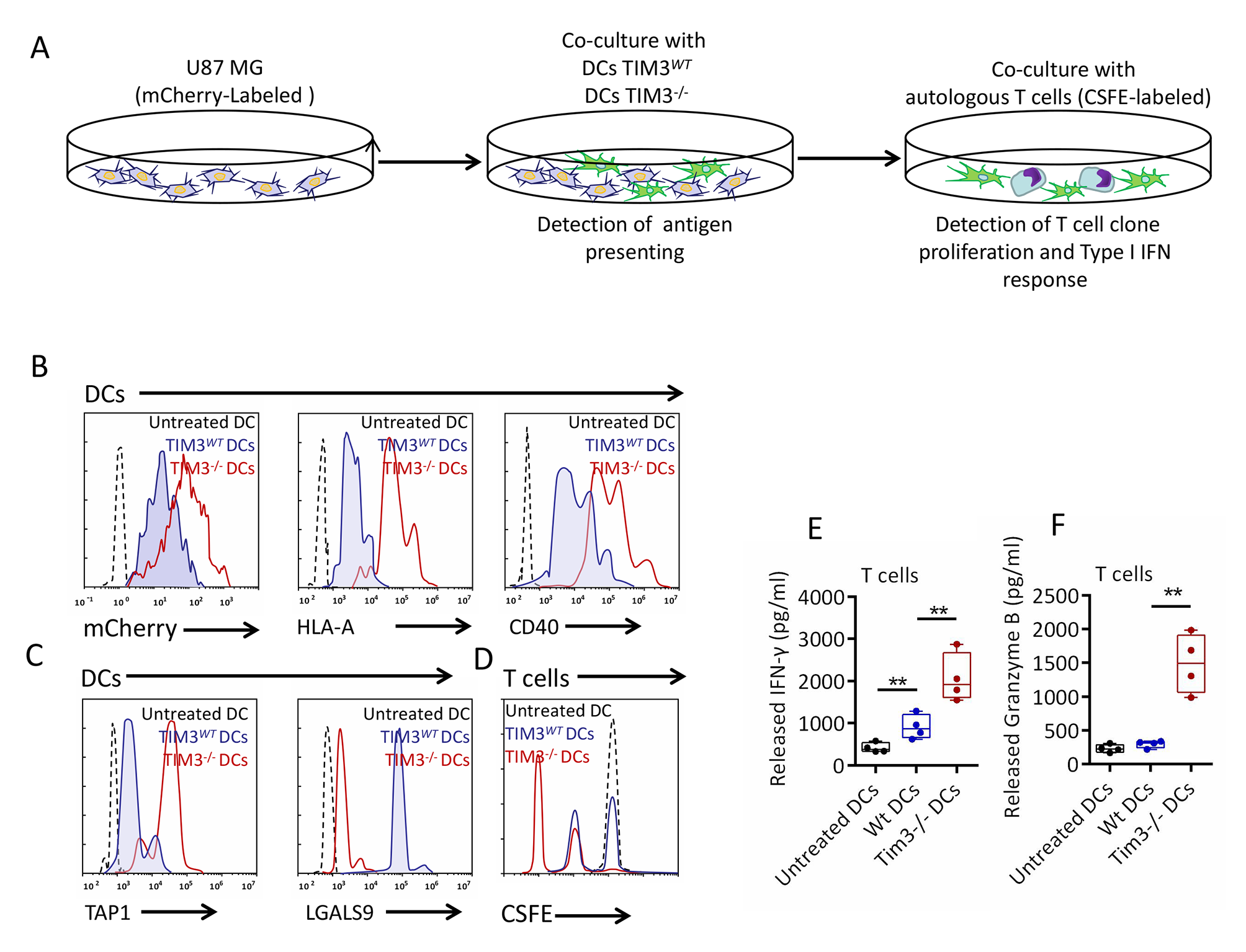

Supplement: Supplementary file 6 — SUPPLEMENTAL Figure 5 [file 41419_2020_3042_MOESM6_ESM.tif]
